# Comparative Evaluation of [68Ga]Ga-DOTA.SA.FAPi and [18F]FDG PET/CT in Metastatic Breast and Lung Cancer: Semiquantitative, Volumetric and Prognostic Assessment

**DOI:** 10.3390/ph19020317

**Published:** 2026-02-14

**Authors:** Sulochana Sarswat, Sanjana Ballal, Madhav Prasad Yadav, Madhavi Tripathi, Prabhat Singh Malik, Sandeep R. Mathur, Frank Rösch, Chandrasekhar Bal

**Affiliations:** 1 Department of Nuclear Medicine, All India Institute of Medical Sciences, New Delhi 110029, India; 2 Department of Medical Oncology, All India Institute of Medical Sciences, New Delhi 110029, India; 3 Department of Pathology, All India Institute of Medical Sciences, New Delhi 110029, India; 4 Department of Chemistry—TRIGA Site, Johannes Gutenberg University, 55099 Mainz, Germany

**Keywords:** [68Ga]Ga-DOTA.SA.FAPi PET/CT, [18F]FDG PET/CT, metastatic breast cancer, lung adenocarcinoma, volumetric PET metrics, progression-free survival

## Abstract

**Objective:** To compare metastatic lesion detection on [68Ga]Ga-DOTA.SA.FAPi and [18F]FDG PET/CT in metastatic breast and lung cancers and to assess the relationship between PET-derived imaging parameters and progression-free survival (PFS). **Methods:** In this prospective dual-cohort study, 45 patients (23 breast cancer, 22 lung adenocarcinoma) underwent paired [68Ga]Ga-DOTA.SA.FAPi and [18F]FDG PET/CT within four weeks. Semiquantitative (SUVmax, SUVmean) and volumetric (MTV, TLG, STV, TLF) PET parameters were measured. Metastatic detection was compared, and correlations with PFS were assessed. **Results:** In breast cancer, [18F]FDG demonstrated higher primary tumor uptake, whereas [68Ga]Ga-DOTA.SA.FAPi showed lower background activity, resulting in higher tumor-to-background ratios for brain and bone metastases. Whole-body volumetric indices (wbTLG, wbTLF) showed strong inverse correlations with PFS. In lung adenocarcinoma, volumetric FAPi-derived parameters (wbTLF, wbSTV) demonstrated modest but significant correlations with PFS. [68Ga]Ga-DOTA.SA.FAPi PET/CT detected more brain metastases than [18F]FDG PET/CT in both cohorts (breast: 15/15 vs. 8/15; lung: 14/14 vs. 4/14). **Conclusions:** [68Ga]Ga-DOTA.SA.FAPi and [18F]FDG PET/CT provide complementary diagnostic and prognostic information. In metastatic breast cancer, FAPi-derived volumetric parameters strongly correlate with PFS and improve detection of brain metastases. In lung adenocarcinoma, [68Ga]Ga-DOTA.SA.FAPi PET/CT offers low background uptake and prognostically relevant stromal metrics. These findings support a potential role for integrating [68Ga]Ga-DOTA.SA.FAPi PET/CT into disease staging, prognostication, and treatment monitoring. This study did not involve prospective assignment to health-related interventions and therefore did not require clinical trial registration.

## 1. Introduction

18F-fluorodeoxyglucose ([18F]FDG) PET/CT is a cornerstone imaging modality in oncology, widely used for staging, treatment response evaluation, and prognostication in various malignancies [1,2,3]. FDG uptake reflects enhanced glycolysis in tumor cells but is not tumor-specific. Its diagnostic accuracy can be confounded by inflammation, infection, post-therapeutic changes, and physiological uptake in organs such as the brain, liver, and myocardium [4]. These limitations may reduce lesion detectability, particularly for small or intracranial metastases.

Fibroblast activation protein (FAP) is selectively expressed on cancer-associated fibroblasts (CAFs) within the tumor microenvironment. Radiolabeled FAP inhibitors (FAPi), including 68Ga- and 18F-labeled derivatives, have demonstrated favorable biodistribution, rapid tumor uptake, and low physiological background, enabling superior tumor-to-background contrast [5,6]. This makes FAPi PET/CT a promising complement or alternative to FDG, particularly for cancers with challenging imaging environments or for detection of lesions in high-background regions such as the brain.

Several head-to-head studies have compared FAPi and [18F]FDG PET/CT across tumor types, reporting improved lesion delineation and higher tumor-to-background ratios (TBRs) with FAPi [7,8,9,10,11,12]. However, prospective data in breast and lung cancer remain limited, despite these malignancies accounting for a substantial portion of the global cancer burden [13,14,15].

In breast cancer, [18F]FDG PET/CT is established for evaluating tumor aggressiveness, molecular subtype, and prognosis. However, physiological uptake and heterogeneous FDG avidity may limit lesion detection. FAPi PET/CT, by targeting stromal activity, may improve volumetric tumor burden assessment, detect occult metastases, and potentially offer prognostic information. In lung cancer, [18F]FDG PET/CT is central to staging and treatment planning. Yet, cerebral metastases are often missed due to high physiological uptake. FAPi PET/CT, with negligible brain background, addresses this limitation. Additionally, FAPi uptake could correlate with specific molecular alterations such as Epidermal Growth Factor Receptor (EGFR) or Anaplastic Lymphoma Kinase (ALK) mutations, providing biologically relevant information.

The present study is a prospective, dual-cohort comparison of Gallium-68-labeled DOTA-squaramide fibroblast activation protein inhibitor ([68Ga]Ga-DOTA.SA.FAPi) PET/CTand [18F]FDG PET/CT in metastatic breast and lung cancers. We aimed to:

1. Compare semiquantitative (SUVmax, SUVmean) and volumetric (MTV, TLG, STV, TLF) PET parameters between tracers;
2. Evaluate metastatic site detection, including brain metastases;
3. Correlate PET-derived parameters with progression-free survival (PFS).

While volumetric PET biomarkers and FAPi imaging have been explored in individual tumor types, prospective comparative data integrating volumetric analysis with survival outcomes across metastatic breast and lung cancers are scarce. Analyzing both breast and lung cancers enables assessment of FAPi and FDG behavior across tumors with differing biological profiles, offering broader insight into tracer performance and prognostic utility.

## 2. Results

**Figure 1:**
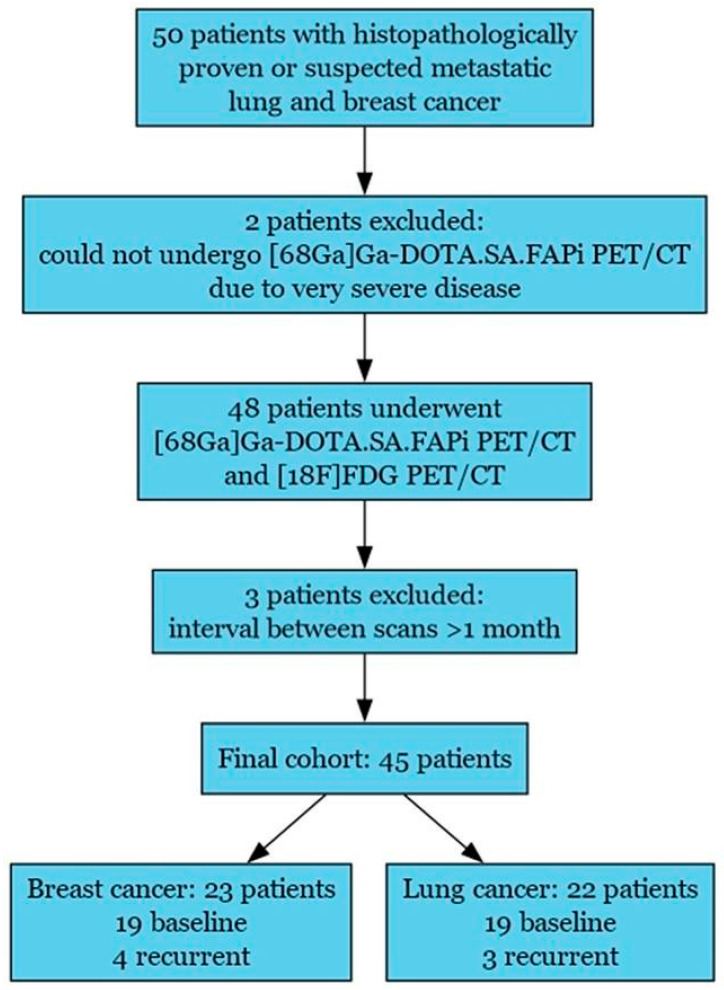
Study flowchart depicting patient enrolment, exclusions, and final cohort composition.

Of 50 screened patients, 45 (23 breast, 22 lung) completed both [68Ga]Ga-DOTA.SA.FAPi and [18F]FDG PET/CT scans within a 4-week interval (Figure 1). Metastatic status was confirmed by histopathology or ≥12 months of clinical–radiological follow-up. No false-negative brain metastases were observed on [68Ga]Ga-DOTA.SA.FAPi PET/CT when compared with CE-MRI.

Comparisons between tracers were done to analyze overall trends in uptake patterns and volumetric tumor burden, which are detailed in the corresponding tables.

### 2.1. Breast Cancer Cohort

#### 2.1.1. Demographics and Imaging Variables in Breast Cancer Cohort

All patients had stage IV disease, with luminal B as the predominant subtype. Progression-free survival (PFS) was analyzed against baseline PET parameters.

No significant differences were observed between [68Ga]Ga-DOTA.SA.FAPi PET/CT and [18F]FDG PET/CT for uptake in primary tumors, lymph nodes, or bone metastases when assessed using SUV-based parameters (Table 1).

#### 2.1.2. Tracer Comparison: SUV and Volumetric Tumor Burden in Breast Cancer

Marked differences were noted in background activity and tumor-to-background contrast. [68Ga]Ga-DOTA.SA.FAPi demonstrated significantly lower physiological up-take in the brain and liver, resulting in substantially higher tumor-to-background ratios for both brain and bone metastases. In contrast, FDG showed significantly higher primary tumor SUV-based uptake and higher whole-body volumetric tumor burden indices (Table 2).

At the lesion level, volumetric FDG metrics exceeded corresponding [68Ga]Ga-DOTA.SA.FAPi parameters, whereas nodal, lung, and liver metastases showed no statistically significant differences between tracers. Despite lower absolute uptake, [68Ga]Ga-DOTA.SA.FAPi consistently demonstrated comparable or higher lesion conspicuity due to reduced background activity.

#### 2.1.3. Detection of Metastases in Breast Cancer

**Figure 2:**
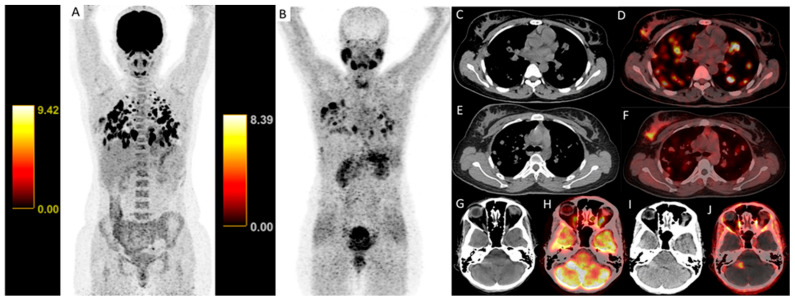
Maximum-intensity projection (MIP) and axial positron emission tomography/computed tomography (PET/CT) images demonstrate differential tracer uptake in a patient with metastatic breast cancer. Panels a and b show whole-body MIP images for [18F]FDG (**A**) and [68Ga]Ga-DOTA.SA.FAPi (**B**), highlighting the primary tumor and multiple metastatic lesions, including lung metastases. Axial computed tomography (CT) images and fused PET/CT images ([18F]FDG PET/CT: (**C**,**G**), (**D**,**H**); [68Ga]Ga-DOTA.SA.FAPi PET/CT: (**E**,**I**); (**F**,**J**) respectively) are shown. Axial brain CT (**I**) and fused PET/CT (**J**) images demonstrate a metastasis in the right cerebellar hemisphere with higher lesion-to-background contrast on [68Ga]Ga-DOTA.SA.FAPi. SUV color bars displayed adjacent to the MIP images indicate the SUV scale used for PET image display and apply to both the MIP and corresponding axial fused images (SUV scale: 0–9.42 for [18F]FDG and 0–8.39 for [68Ga]Ga-DOTA.SA.FAPi). Abbreviations: MIP, maximum-intensity projection; PET, positron emission tomography; CT, computed tomography.

[68Ga]Ga-DOTA.SA.FAPi detected significantly more brain metastases than FDG (Figure 2), a finding confirmed by McNemar’s test (*p* = 0.016).

#### 2.1.4. Patient-Based Comparison in Breast Cancer

On a patient-based analysis, [18F]FDG showed moderate sensitivity but perfect specificity for brain metastasis detection, while lesion-based analysis demonstrated a substantially lower detection rate. Detection of lymph node, bone, liver, and lung metastases showed high concordance between [68Ga]Ga-DOTA.SA.FAPi and [18F]FDG PET/CT.

#### 2.1.5. Luminal Subtype Variation in Tracer Uptake

Subtype analysis revealed no significant differences in tracer uptake across luminal subtypes, although interpretation was limited by small subgroup sizes.

### 2.2. Lung Cancer Cohort

#### 2.2.1. Demographics and Imaging Variables in Lung Cancer Cohort

All lung cancer patients had adenocarcinoma histology, with a high prevalence of EGFR mutations and a smaller proportion of ALK rearrangements. PFS was longer compared with the breast cancer cohort (Table 3).

#### 2.2.2. Tracer Comparison: SUV and Volumetric Tumor Burden in Lung Cancer

Primary tumor SUV-based uptake did not differ significantly between [68Ga]Ga-DOTA.SA.FAPi and [18F]FDG PET/CT. As in breast cancer, the former demonstrated markedly lower background uptake in the brain and liver, resulting in significantly higher tumor-to-background ratios for intracranial metastases (Table 4). Whole-body volumetric indices were broadly comparable between tracers.

#### 2.2.3. Detection of Metastases in Lung Cancer

**Figure 3:**
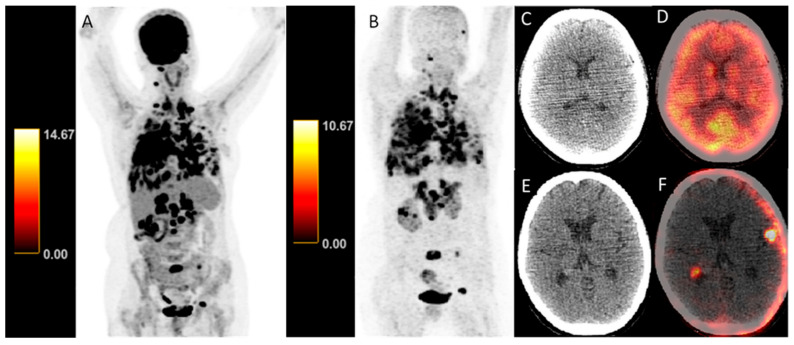
Maximum-intensity projection (MIP) and axial PET/CT images demonstrate differential tracer uptake in a patient with metastatic lung cancer. Panels A and B show whole-body MIP images for [18F]FDG (**A**) and [68Ga]Ga-DOTA.SA.FAPi (**B**), highlighting primary tumor and multiple metastatic lesions in lungs, lymph nodes, and bones. Axial CT (**C**,**E**) and fused PET/CT (**D**,**F**) images depict intracranial metastases: c and d correspond to [18F]FDG CT and fused PET/CT, whereas e and f correspond to [68Ga]Ga-DOTA.SA.FAPi CT and fused PET/CT, demonstrating higher lesion-to-background contrast with [68Ga]Ga-DOTA.SA.FAPi SUV color bars displayed adjacent to the MIP images indicate the SUV scale used for PET image display and apply to both the MIP and corresponding axial fused images (0–14.67 for [18F]FDG and 0–10.67 for [68Ga]Ga-DOTA.SA.FAPi). Abbreviations: MIP, maximum-intensity projection; PET, positron emission tomography; CT, computed tomography.

All brain metastases were detected on [68Ga]Ga-DOTA.SA.FAPi PET/CT, whereas [18F]FDG PET/CT demonstrated low sensitivity on both patient-based and lesion-based analyses (Figure 3). [18F]FDG PET/CT missed the majority of intracranial lesions but retained perfect specificity. Detection of bone, lymph node, liver, and lung metastases showed high concordance between tracers, with [68Ga]Ga-DOTA.SA.FAPi identifying additional lesions in selected cases.

#### 2.2.4. Molecular Subgroup Analysis in Lung Cancer

In the lung cancer cohort, EGFR-mutated tumors demonstrated significantly higher whole-body stromal tumor volume on [68Ga]Ga-DOTA.SA.FAPi PET/CT, while ALK-positive tumors showed higher primary stromal tumor volume (Table 5).

### 2.3. Correlation Analysis Between [18F]FDG and [68Ga]Ga-DOTA.SA.FAPi PET/CT Parameters

#### 2.3.1. Tracer-Specific Inter-Parameter Relationship in Breast Cancer

**Figure 4:**
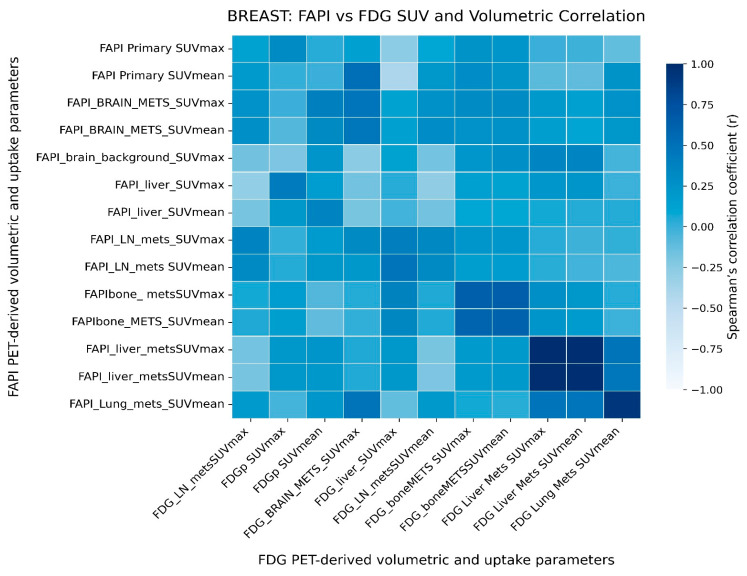
Heatmap showing Spearman’s correlation between comparing [18F]FDG and [68Ga]Ga-DOTA.SA.FAPi PET/CT-derived volumetric and uptake parameters in the breast cancer cohort. Color intensity represents the strength and direction of correlation (Spearman’s r), as indicated by the color scale.

Spearman correlation heatmaps demonstrated distinct association patterns for each tracer. Volumetric parameters derived from [68Ga]Ga-DOTA.SA.FAPi PET/CT exhibited strong internal concordance and showed moderate correlations with the corresponding metrics on [18F]FDG PET/CT (Figure 4).

#### 2.3.2. Tracer-Specific Inter-Parameter Relationship in Lung Cancer

**Figure 5:**
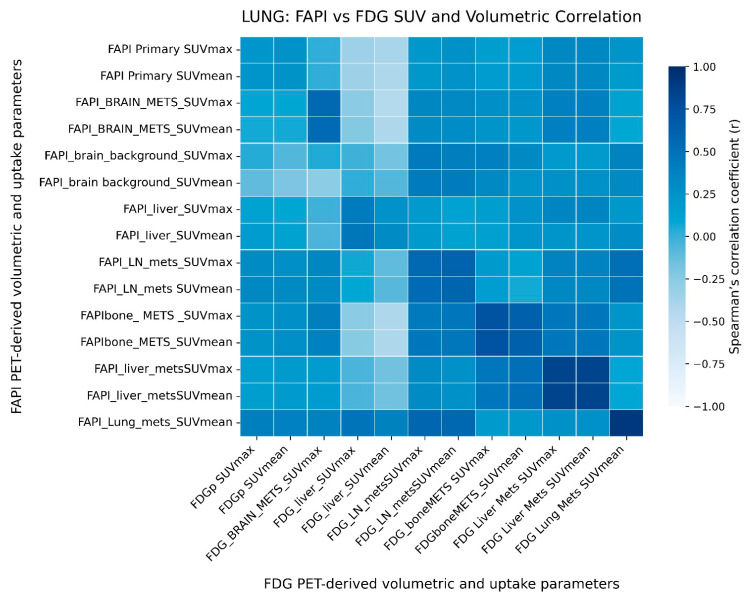
Heatmap showing Spearman’s correlation between [18F]FDG and [68Ga]Ga-DOTA.SA.FAPi PET/CT-derived volumetric and uptake parameters in the lung cancer cohort. Color intensity represents the strength and direction of correlation (Spearman’s r), as indicated by the color scale.

In lung cancer, correlations between [68Ga]Ga-DOTA.SA.FAPi and [18F]FDG parameters were weaker and more heterogeneous, indicating divergent biological behavior (Figure 5).

### 2.4. Correlation of PET/CT Parameters with PFS (Progression Free Survival)

#### 2.4.1. Prognostic Correlation in Breast Cancer

In the breast cancer cohort, volumetric PET parameters derived from both [68Ga]Ga-DOTA.SA.FAPi PET/CT and [18F]FDG PET/CT demonstrated strong inverse correlations with PFS, with whole-body tumor burden indices emerging as the strongest predictors (Table 6). SUV-based parameters from both tracers showed no prognostic association.

#### 2.4.2. Prognostic Correlation in Lung Cancer

In lung cancer, prognostic correlations were weaker. Only whole-body stromal indices derived from [68Ga]Ga-DOTA.SA.FAPi PET/CT showed significant inverse associations with PFS, whereas lesion-level volumetric metrics and SUV-based parameters were not predictive (Table 7).

## 3. Discussion

This prospective dual-cohort study provides a head-to-head comparison of [68Ga]Ga-DOTA.SA.FAPi PET/CT and [18F]FDG PET/CT in metastatic breast and lung cancer, highlighting complementary strengths of each tracer and the prognostic value of volumetric parameters.

In breast cancer, FDG demonstrated higher primary tumor SUVs, consistent with its sensitivity to glycolytic metabolism [1]. [68Ga]Ga-DOTA.SA.FAPi uptake in primary tumors was significantly lower (median SUVmax 4.30 vs. 11.84, *p* < 0.001), reflecting its distinct biological target, fibroblast activation protein expressed by cancer-associated fibroblasts (CAFs) in the tumor stroma. This discrepancy underscores that FDG primarily reflects tumor cell metabolic activity, whereas FAPi represents stromal activity. Consequently, volumetric indices such as pSTV, wbSTV, and wbTLF were consistently smaller than their FDG equivalents, likely reflecting lower stromal density within the primary mass. Prior studies have reported similar findings; Giesel et al. and Xiong M et al. documented complementary detection patterns between FAPi and FDG, emphasizing tumor heterogeneity and the differential distribution of metabolic and stromal activity [7,8]. Kuten et al. also observed higher FDG SUVmax in primary breast lesions, whereas FAPi highlighted metastatic sites with lower FDG avidity [10].

Importantly, in our breast cancer cohort, volumetric parameters, particularly wbTLG and wbTLF, correlated strongly with PFS, whereas SUV-based metrics did not. Whole-body TLG (ρ = −0.688, *p* < 0.001) and wbTLF (ρ = −0.674, *p* < 0.001) were the most robust predictors, followed by wbMTV and wbSTV. This observation aligns with prior evidence suggesting that volumetric PET indices integrate total tumor burden and biological heterogeneity, offering superior prognostic insight compared with focal SUV measurements [9,10]. The stronger correlation of stromal-targeted [68Ga]Ga-DOTA.SA.FAPi volumetrics with PFS in breast versus lung cancer may reflect differences in tumor biology: breast metastases often have a more pronounced stromal component and heterogeneous [18F]FDG avidity, whereas lung adenocarcinomas may exhibit more uniform glycolytic activity with less stromal contribution. Clinically, this suggests that FAPi-derived volumetrics could complement FDG in risk stratification and therapy monitoring, particularly for patients with widespread metastatic disease or indolent tumors with low glycolytic activity.

A key clinical advantage of [68Ga]Ga-DOTA.SA.FAPi PET/CT was its superior detection of brain metastases. Low physiological cerebral uptake facilitated high tumor-to-background ratios, revealing metastases that [18F]FDG PET/CT missed. In our breast cancer subset, the former detected brain lesions in patients who were falsely negative on [18F]FDG PET/CT (sensitivity 100% vs. 53.3%), supporting its potential clinical utility in comprehensive staging. Accurate intracranial lesion detection is critical for therapy planning, including stereotactic radiotherapy and systemic therapy decisions, and may improve patient outcomes.

Subtype analysis in breast cancer revealed no significant differences in tracer uptake across luminal A, B, and C groups, indicating that FAPi and FDG signal predominantly reflects tumor biology rather than molecular subtype. However, small numbers in luminal A and C limited definitive conclusions; larger studies are needed to evaluate subtype-specific tracer dynamics.

In lung cancer, tracer uptake was broadly comparable between [68Ga]Ga-DOTA.SA.FAPi and [18F]FDG PET/CT, with median primary tumor SUVmax values of 7.32 (5.54–10.85) and 9.77 (6.11–18.63) respectively, reflecting heterogeneous stromal composition in adenocarcinomas [7]. Nevertheless, [68Ga]Ga-DOTA.SA.FAPi provided marked background suppression in the brain and liver, enhancing TBR and lesion conspicuity in these challenging regions. Whole-body total lesion FAP uptake (TLF) was significantly correlated with progression-free survival (PFS; ρ = −0.600, *p* = 0.0032), while whole-body stromal tumor volume (wbSTV) also showed a significant moderate correlation (ρ = −0.429, *p* = 0.046), suggesting that stromal tumor burden may serve as a prognostic biomarker independent of glycolytic activity. EGFR- and ALK-mutated tumors demonstrated higher FAPi STV, hinting at a possible link between oncogenic driver mutations and stromal activation. This warrants further investigation, as it may inform personalized imaging and targeted therapy strategies.

Across both cohorts, whole-body volumetric indices (wbSTV and wbTLF) showed positive correlations with disease burden and PFS. These associations were stronger in breast cancer, consistent with its higher stromal content and diffuse FAPi uptake, whereas lung cancer demonstrated comparatively weaker correlations due to greater heterogeneity and lower stromal contribution.

Strengths of the current study include the paired intra-patient design, comprehensive semiquantitative and volumetric PET analysis, and incorporation of survival outcomes. Limitations include modest sample size per molecular subgroup, single-center recruitment, and absence of overall survival data. The study was not powered for molecular subgroup analysis, particularly for less common breast subtypes and ALK-positive lung cancer. Although disease progression within the scan interval could theoretically influence lesion burden, the short median inter-scan interval (10 days) likely minimized any clinically meaningful bias.

## 4. Materials and Methods

### 4.1. Study Design and Patient Selection

This was a prospective, single-center observational imaging study. No therapeutic or diagnostic interventions were assigned as part of the study protocol, and the study does not meet the World Health Organization definition of a clinical trial. The study enrolled adults aged 18–75 years with biopsy-proven or clinically suspected metastatic breast or lung carcinoma. Histopathological confirmation of metastatic disease was available in 10 patients. Histopathological diagnosis of the primary tumor was available for all patients, as this constituted an inclusion criterion. In the remaining cases, metastatic lesions were confirmed based on clinicoradiological follow-up, including serial imaging and clinical progression, in accordance with standard oncologic practice.

Ethical approval was obtained from the institutional review board of the All India Institute of Medical Sciences, New Delhi (approval code: IEC-PG 77/27.02.2020, RT-30/23.04.2020; approval date: 2 May 2020), and was conducted in accordance with the Declaration of Helsinki. Written informed consent was obtained from all participants for participation and the publication of anonymized data. Exclusion criteria included inability to undergo both [68Ga]Ga-DOTA.SA.FAPi PET/CT and [18F]FDG PET/CT (non-contrast) scans within an interval of 4 weeks between scans, or administration of any intervening therapy. Although a maximum interval of up to 4 weeks between the two PET/CT scans was permitted, this represented an extreme upper limit; the median interval between scans was 10 days.

### 4.2. Radiotracer Synthesis and PET/CT Acquisition

[68Ga]Ga-DOTA.SA.FAPi was synthesized by eluting 68GaCl_3_ solution (~925 MBq), which was added to a reaction vial containing DOTA.SA.FAPi and ammonium acetate and heated at 90 °C for 10 min. The radiolabeled product was purified using a Sep-Pak C18 cartridge with 70% ethanol, followed by 10 mL of normal saline. Fluorine-18 fluorodeoxyglucose ([18F]FDG) was synthesized according to an established protocol. Radiochemical purity was >95% for both radiotracers.

Whole-body PET/CT scans were performed 60 min after intravenous injection using a GE Discovery 710 scanner (GE Medical Systems, Waukesha, WI, USA)with the following administered activities:

- [68Ga]Ga-DOTA.SA.FAPi: 0.05–0.06mCi/kg (1.81–2.18 MBq/kg)
- [18F]FDG: 0.10–0.12 mCi/kg (3.70–4.44 MBq/kg)

Patients fasted for at least 6 h prior to [18F]FDG PET/CT. Blood glucose levels were measured to ensure levels < 150 mg/dL. PET/CT images were reconstructed using iterative algorithm optimal subset expectation maximization (OSEM) with standard corrections for attenuation, scatter, and random, ensuring reproducibility across scanners.

### 4.3. Image Analysis

PET/CT images were independently reviewed by two experienced nuclear medicine physicians blinded to clinical outcomes. Lesions were categorized as primary, nodal, or distant metastases. Semiquantitative parameters included SUVmax and SUVmean. Volumetric parameters were calculated as:

- MTV: Metabolic Tumor Volume (FDG);
- TLG: Total Lesion Glycolysis (FDG);
- STV: Stromal Tumor Volume (FAPi);
- TLF: Total Lesion FAP expression (FAPi).

Image segmentation and quantification were performed on a GE Advantage Work-station (GE Healthcare, Waukesha, WI, USA; software version AW 4.6). Volumetric parameters, including STV, MTV, TLF, and TLG, were derived using a semi-automatic 42% SUVmax threshold to delineate metabolically active tumor volumes, as commonly applied in oncologic PET volumetry.

Whole-body indices were calculated as the sum of all individual lesions. Regions of interest (ROIs) were delineated using PERCIST 1.0 recommendations.

### 4.4. Confirmation of Metastatic Status

All the patients were followed up for ≥12 months (median follow up period 18 months). Contrast-enhanced MRI (CE-MRI) of the brain was used as the reference standard for confirmation of all brain metastases. All intracranial lesions detected on PET/CT were correlated with CE-MRI findings. Lesions showing tracer uptake on PET/CT were considered true positives only if corresponding metastatic lesions were identified on CE-MRI.

### 4.5. Statistical Analysis

Data were tested for normality using the Shapiro–Wilk test. Continuous variables are presented as mean ± standard deviation (SD) for normally distributed data and median (interquartile range, IQR) for skewed data. Paired comparisons between FAPi and FDG parameters were performed using paired t-tests (for normal data) or Wilcoxon signed-rank tests (for non-normal data).

Diagnostic performance for metastatic detection (sensitivity, specificity, positive predictive value (PPV), negative predictive value (NPV)) was calculated on patient- and lesion-based analyses. Associations between PET-derived parameters and progression-free survival (PFS) were assessed using Spearman’s rank correlation coefficient, as several imaging variables were non-normally distributed and the relationship between volumetric PET metrics and survival outcomes was not assumed to be linear. Spearman correlation was therefore selected as a robust non-parametric method appropriate for skewed data, and correlation heatmaps were generated to visualize inter-parameter relationships. Correlation analyses were exploratory; no correction for multiple comparisons was applied due to the hypothesis-generating nature of the study. Statistical significance was defined as *p* < 0.05. Analyses were performed using SPSS version 28.0 (IBM Corp., Armonk, NY, USA).

### 4.6. Treatment and Prognosis

Treatment was administered according to molecular subtype in line with contemporary breast cancer management guidelines. Hormone receptor–positive patients received endocrine therapy with or without targeted agents, HER2-positive patients were treated with anti-HER2–directed therapy in combination with chemotherapy, and those with triple-negative breast cancer (TNBC) received platinum-based chemotherapy.

For lung cancer, similarly, the treatment was focused on EGFR/ALK mutation targeted drugs, when positive; otherwise, patients received Platinum-based chemotherapy.

## 5. Conclusions

This prospective comparative study demonstrates that both [68Ga]Ga-DOTA.SA.FAPi PET/CT and [18F]FDG PET/CT provide complementary diagnostic and prognostic information in metastatic breast and lung cancers. In breast cancer, whole-body volumetric indices, particularly wbTLG and wbTLF, showed strong associations with progression-free survival, and [68Ga]Ga-DOTA.SA.FAPi PET/CT demonstrated superior detection of brain metastases. In lung cancer, [68Ga]Ga-DOTA.SA.FAPi PET/CT exhibited diagnostic performance comparable to [18F]FDG PET/CT, with the additional advantages of lower background activity and stromal-based volumetric parameters that were prognostically informative. These findings support a disease-specific role for [68Ga]Ga-DOTA.SA.FAPi PET/CT and indicate that FAPi-derived volumetric metrics may enhance risk stratification over conventional semiquantitative measures. Larger multicenter studies are warranted to validate these observations and to further explore the integration of FAPi-based volumetric biomarkers into clinical decision-making algorithms.

## Figures and Tables

**Table 1:** Breast cancer cohort clinical and imaging variables.

Variable	Summary	
Parameter	(Mean ± SD)	
Age (years)	46.78 ± 10.69	
Weight (kg)	55.92 ± 11.43	
Tumor size (cm)	4.18 ± 1.36	
PFS (days)	242.61 ± 102.80	
Outcome, n (%)	Progression Stable Partial Response Complete Response Death	15 (65.2) 3 (13.0) 2 (8.7) 2 (8.7) 1 (4.3)
Luminal subtypes n (%)	A	5 (21.74)
	B	13 (56.56)
	C	5 (21.74)
Imaging parameters (Median/IQR or Mean ± SD)	[68Ga]Ga-DOTA.SA.FAPi PET/CT	[18F]FDG PET/CT
Primary SUVmean	3.71 (2.99–4.76)	4.68 (3.57–5.67)
Primary TBR	4.18 ± 2.63	3.98 (3.18–6.59)
LN mets SUVmax	3.88 (2.21–5.49)	5.99 (3.35–9.44)
LN mets SUVmean	2.85 (1.40–4.65)	4.21 (1.99–6.38)
LN mets TBR	2.43 (1.23–4.58)	2.58 (1.06–4.01)
Bone mets SUVmax	5.22 ± 4.72	4.46 ± 4.31
Bone mets SUVmean	3.89 ± 3.58	3.31 ± 3.23

Continuous variables are presented as Mean ± SD if normally distributed, or Median [IQR] if skewed. Abbreviations: PFS: progression free survival, TBR: tumor-to-background ratio (with liver as reference), mets: metastasis, LN: lymph node, SUV: standardized uptake value. Only parameters without statistically significant differences between [68Ga]Ga-DOTA.SA.FAPi and [18F]FDG PET/CT are presented; hence, *p*-values are not shown.

**Table 2:** Comparison of [68Ga]Ga-DOTA.SA.FAPi and [18F]FDG PET/CT parameters in breast cancer patients.

Parameter	[68Ga]Ga-DOTA.SA.FAPi PET/CT	[18F]FDG PET/CT	*p* Value
pSTV/pMTV	211.08 ± 101.62	237.87 ± 112.91	<0.001 **
wbSTV/wbMTV	1030.48 (612.11–1765.33)	1332.75 (765.13–2248.90)	<0.001 **
wbTLF/wbTLG	1396.39 (924.43–1999.10)	1994.85 (1249.18–2835.70)	<0.001 **
Brain background SUVmax	0.52 ± 0.16	7.43 (6.00–10.36)	<0.001 **
pSUVmax	4.30 (3.13–9.45)	11.84 ± 5.13	<0.001 **
TBR (brain metastases)	5.17 (3.12–11.07)	0.78 ± 0.88	<0.001 **
Liver SUVmax	1.57 ± 0.62	2.19 (1.79–3.41)	<0.001 **
TBR (bone metastases)	3.10 (0.00–6.05)	1.69 (0.00–2.76)	0.016 *
pTLF/pTLG	837.60 (473.09–1144.29)	1144.22 ± 657.56	0.018 *
TBR (liver metastases)	0.00 (0–1.58)	0.00 (0–1.11)	0.028 *

Continuous variables are presented as Mean ± SD if normally distributed, or Median [IQR] if skewed. Abbreviations: p—primary lesion, wb—whole body, MTV—metabolic tumor volume, STV—stromal tumor volume, TLG—total lesion glycolysis, TLF—total lesion FAP expression, TBR—tumor-to-background ratio (liver as reference). Paired comparisons between [68Ga]Ga-DOTA.SA.FAPi and [18F]FDG parameters were performed using the paired *t*-test for normally distributed variables and the Wilcoxon signed-rank test for skewed variables. * *p* < 0.05; ** *p* < 0.01.

**Table 3:** Lung cancer cohort clinical and imaging variables.

Variable	Summary	
Clinical and molecular parameters	Mean ± SD/Median (IQR)	
Age (years)	54.64 ± 12.14	
Weight (kg)	58.50 (53.00–66.12)	
ECOG score	2.59 ± 0.91	
Serum LDH (U/L)	322.0 (250.0–1078.8)	
PFS (days)	534.8 ± 261.7	
	Male	14 (63.6)
Gender, n (%)		
	Female	8 (36.4)
	Yes	15 (68.2)
Smoking, n (%)		
	No	7 (31.8)
	Positive	3 (13.6)
ALK mutation, n (%)		
	Negative	19 (86.4)
	Positive	11 (50.0)
EGFR mutation, n (%)		
	Negative	11 (50.0)
Imaging parameters Mean ± SD/Median (IQR)	[68Ga]Ga-DOTA.SA.FAPi PET/CT	[18F]FDG PET/CT
Primary TBR	3.50 (1.99–6.71)	3.75 (1.92–5.80)
pSTV (mL)	89.27 ± 53.87	39.05 (22.62–70.30)
pTLF/pTLG	415.36 ± 199.37	314.81 (139.01–778.13)
wbSTV (mL)	326.93 ± 143.96	303.50 (187.29–635.13)
wbTLF/wbTLG	1034.18 ± 388.45	663.32 (399.40–1439.63)
LN mets SUVmax	6.30 (3.03–10.76)	6.99 (3.00–11.41)
LN mets SUVmean	4.57 (2.07–8.15)	4.61 (1.81–7.68)

Only parameters without statistically significant differences between [68Ga]Ga-DOTA.SA.FAPi and [18F]FDG PET/CT are presented; *p* values are therefore not shown. Continuous variables are presented as Mean ± SD if normally distributed, or Median [IQR] if skewed. Abbreviations: PFS, progression-free survival; p, primary lesion; TBR, tumor-to-background ratio (liver as reference); mets, metastases; LN, lymph node; MTV, metabolic tumor volume; STV, stromal tumor volume; TLG, total lesion glycolysis; TLF, total lesion FAP expression.

**Table 4:** Comparison of [68Ga]Ga-DOTA.SA.FAPi and [18F]FDG PET/CT parameters in lung cancer patients.

Metric	[68Ga]Ga-DOTA.SA.FAPi	[18F]FDG PET/CT	*p* Value
Primary SUVmax	7.32 (5.54–10.85)	9.77 (6.11–18.63)	0.063
Primary SUVmean	5.51 (4.23–8.34)	7.41 (3.92–12.65)	0.079
Brain background SUVmax	0.73 ± 0.26	7.35 (6.53–10.48)	<0.001 **
TBR (brain metastases)	3.62 (2.19–5.09)	0.00 (0.00–0.00)	<0.001 **
Liver SUVmax	2.18 ± 0.85	3.19 ± 1.10	<0.001 **
Liver SUVmean	1.37 (1.15–1.75)	1.98 (1.83–2.87)	0.002 **

Continuous variables are presented as Mean ± SD if normally distributed, or Median [IQR] if skewed. Paired comparisons were performed using the paired *t*-test for normally distributed variables and the Wilcoxon signed-rank test for skewed variables. Abbreviations: TBR, tumor-to-background ratio. ** *p* < 0.01.

**Table 5:** PET parameters showing significant difference by mutation status in lung cancer.

Variable	Group	Group Sizes (Positive, Negative)	*p*-Value
pSTV	EGFR mutation	11, 11	0.036 *
pSTV	ALK mutation	19, 3	0.030 *
wbSTV	EGFR mutation	11, 11	0.036 *

p: primary, STV: stromal tumor volume, wb: whole-body. Comparisons were performed using the Mann–Whitney U test, * *p* value < 0.05.

**Table 6:** Spearman correlation between PET-derived parameters and progression-free survival in breast cancer.

Variable	Spearman ρ	*p*-Value
wbTLG	−0.688	0.0003 **
pTLG	−0.686	0.0003 **
wbTLF	−0.674	0.0004 **
wbMTV	−0.618	0.0017 *
wbSTV	−0.591	0.003 **
pSTV	−0.558	0.0057 **
pMTV	−0.558	0.0057 **
pTLF	−0.513	0.012 *

Abbreviations: wb, whole body; p, primary tumor; TLG, total lesion glycolysis; TLF, total lesion FAP expression; MTV, metabolic tumor volume; STV, stromal tumor volume. Negative Spearman ρ values indicate an inverse correlation with progression-free survival. * *p* < 0.05; ** *p* < 0.01.

**Table 7:** Spearman correlation between PET-derived parameters and progression-free survival in lung cancer.

Variable	Spearman’s ρ	*p* Value
wbTLF	−0.600	0.0032 **
wbSTV	−0.429	0.046 *
pSTV	−0.312	0.158
pTLF	−0.289	0.192
pMTV	−0.141	0.530
wbTLG	−0.062	0.785
wbMTV	0.007	0.976

Abbreviations: wb—whole-body; p—primary tumor; TLG—total lesion glycolysis; TLF—total lesion FAPi uptake; MTV—metabolic tumor volume; STV—stromal tumor volume. * *p* < 0.05; ** *p* < 0.01.

## Data Availability

The data presented in this study are available from the corresponding author upon reasonable request. The data are not publicly available due to patient privacy and ethical restrictions.

## Abbreviations

The following abbreviations are used in this manuscript:

ALK	Anaplastic Lymphoma Kinase
CAF	Cancer-Associated Fibroblast
CT	Computed Tomography
ECOG	Eastern Cooperative Oncology Group
EGFR	Epidermal Growth Factor Receptor
FAP	Fibroblast Activation Protein
FAPi	Fibroblast Activation Protein Inhibitor
FDG	Fluorodeoxyglucose
IEC	Institutional Ethics Committee
IQR	Interquartile Range
LDH	Lactate Dehydrogenase
LN	Lymph Node
MIP	Maximum-Intensity Projection
MTV	Metabolic Tumor Volume
OSEM	Ordered Subset Expectation Maximization
PET	Positron Emission Tomography
PERCIST	PET Response Criteria in Solid Tumors
PFS	Progression-Free Survival
PPV	Positive Predictive Value
ROI	Region of Interest
SD	Standard Deviation
STV	Stromal Tumor Volume
TLF	Total Lesion FAP
TLG	Total lesion glycolysis
